# Supplementary material for: Testing electron–phonon coupling for the superconductivity in kagome metal CsV3Sb5
Source: Nat Commun. 2023 Apr 7;14:1945. doi: 10.1038/s41467-023-37605-7 (PMC10082024; doi:10.1038/s41467-023-37605-7)
Supplement: Supplementary file 1 — Supplementary information [file 41467_2023_37605_MOESM1_ESM.pdf]

Supplementary Information for
Testing Electron-phonon Coupling for the Superconductivity in Kagome Metal CsV_3Sb_5

Yigui Zhong[1,†], Shaozhi Li[2,†], Hongxiong Liu[3,†], Yuyang Dong[1], Kohei Aido[1], Yosuke Arai[1], Haoxiang Li[2,4], Weilu Zhang[1,5], Youguo Shi[3], Ziqiang Wang[6], Shik Shin[1,7], H. N. Lee[2], H. Miao[2,*], Takeshi Kondo[1,8,*], Kozo Okazaki[1,8,9,*]

[1]Institute for Solid State Physics, The University of Tokyo, Kashiwa, Chiba 277-8581, Japan

[2]Material Science and Technology Division, Oak Ridge National Laboratory, Oak Ridge, Tennessee 37831, USA

[3]Beijing National Laboratory for Condensed Matter Physics and Institute of Physics, Chinese Academy of Sciences, 100190, Beijing, China

[4]Advanced Materials Thrust, The Hong Kong University of Science and Technology (Guangzhou), 511453, Guangzhou, Guangdong, China

[5]Department of Engineering and Applied Sciences, Sophia University, Tokyo 102-8554, Japan

[6]Department of Physics, Boston College, Chestnut Hill, Massachusetts 02467, USA

[7]Office of University Professor, The University of Tokyo, Kashiwa, Chiba 277-8581, Japan

[8]Trans-scale Quantum Science Institute, The University of Tokyo, Bunkyo, Tokyo 113-0033, Japan

[9]Material Innovation Research Center, The University of Tokyo, Kashiwa, Chiba 277-8561, Japan

[†]These authors contributed equally.

[*]*Corresponding author: miaoh@ornl.gov, kondo1215@issp.u-tokyo.ac.jp, okazaki@issp.u-tokyo.ac.jp*

1. Bare band assumption and extraction of the single particle self-energy

It is well-established that the single particle self-energy $\Sigma(\omega) = Re\Sigma + i * Im\Sigma$ can be extracted from the experimental ARPES data following that[1]:

$$Re\Sigma = E_{\boldsymbol{k}} - \varepsilon_{\boldsymbol{k}} \quad (1),$$

$$Im\Sigma = |v_0| * \text{FWHM}/2 \quad (2),$$

where the $E_{\boldsymbol{k}}$ is the experimental band dispersion determined by the peak of the MDCs; the $\varepsilon_{\boldsymbol{k}}$is the bare band with a bare velocity v_0; FWHM is the full-width-at-half-maximum of the fitted MDCs. In this manuscript, since the α and β bands are isolated in momentum space, we fit the MDCs based on a standard single-peak Lorentzian function to extract the peaks and FWHMs.

The renormalization induced by the electron-phonon coupling (EPC) is rapidly decayed when approaching the binding energy (E_B) far beyond the phonon frequency, the bare band $\varepsilon_{\boldsymbol{k}}$ can be assumed as a linear dispersion connecting a large E_B and the Fermi level (E_F), namely $\varepsilon_{\boldsymbol{k}} = v_0 \hbar \boldsymbol{k}$. In this manuscript, we choose a linear dispersion connecting the E_B~100 meV and the E_F as the bare band. With this assumption, the self-energy presented in the main text is extracted following Eqs. (1) and (2).

Since the imaginary-part self-energy ImΣ(ω) is calculated from the FWHM of the MDCs, it is important to estimate the influence from the k_z broadening. This broadening depends on details of the band dispersion along k_z as well as the electron coherence along the surface normal. DFT calculations predicted a neglectable k_z-dependence of the β band near E_F (ref[2]). For the α band, it is confirmed by the photon energy dependent ARPES measurements[3] that its k_z-dependence is also neglectable within ~200 meV below E_F. Since our linewidth analysis from the ARPES spectra was performed within 100 meV below E_F, the k_z broadening is considered to have a minimal effect.

2. Subtraction of the backgrounds in the extracted ImΣ(ω)

Assuming that the microscopic scattering processes are independent, the total imaginary-part self-energy derived from the FWHM of the fitted MDCs for a non-magnetic metal is given by ImΣ = $Im\Sigma_{ep} + Im\Sigma_{ele} + Im\Sigma_{imp}$ (refs[4,5]). Here $Im\Sigma_{ep}$ is contributed from the electron-phonon couplings, which has a characteristic step feature at the energy of the coupled phonon mode. For E_B much higher than this step energy, $Im\Sigma_{ep}$ is nearly constant. For the electron-electron correlation part, $Im\Sigma_{ele}$ is proportional to ω^{α} (e.g. α = 2 for a Fermi liquid and α = 1 for a marginal Fermi liquid,

etc.). Besides these main energy-dependent self-energy parts, electron-impurity scattering will give rise to energy-independent self-energy $\mathrm{Im}\Sigma_{imp}$.

To extract $\mathrm{Im}\Sigma_{ep}$, one needs to subtract a background of $\mathrm{Im}\Sigma_{others} = \mathrm{Im}\Sigma_{ele} + \mathrm{Im}\Sigma_{imp}$ from raw $\mathrm{Im}\Sigma$ derived from the FWHMs of the fitted MDCs (Eq. (2)). Based on above discussions, it is reasonable to assume $\mathrm{Im}\Sigma_{others}= C_0 + C_1\omega^2$. We evaluate the parameter C_1 from a parabolic fit to $\mathrm{Im}\Sigma$ at $\omega = E_B > 40$ meV and assign $C_0 = \mathrm{Im}\Sigma_0 = \mathrm{Im}\Sigma(E_B=0)$. As examples, in Figs. S1a-b, we demonstrate the subtraction of $\mathrm{Im}\Sigma_{others}$ for the α and β bands of CsV_3Sb_5. It turns out that electron-electron interactions, as the yellow shadows shown in Figs. S1a-b, have a minor contribution to the total $\mathrm{Im}\Sigma$. Quantitively, it is smaller than ~2 meV with E_B ~ [0, 60] meV.

3. Examining the consistency of self-energy via Kramers-Kronig relation

The self-energy $\Sigma(\omega)$ induced by electron-boson interactions as a complex function, its real prat and imaginary parts are not independent but associated together by the Kramers-Kronig (K-K) relation (see Eq. (3)), which helps to check the consistency of the extracted self-energy[4-6].

$$\begin{aligned}\mathrm{Re}\Sigma(\omega) &= \frac{1}{\pi} P \int_{-\infty}^{+\infty} \frac{\mathrm{Im}\Sigma(\omega')}{\omega' - \omega} d\omega' \\ \mathrm{Im}\Sigma(\omega) &= -\frac{1}{\pi} P \int_{-\infty}^{+\infty} \frac{\mathrm{Re}\Sigma(\omega')}{\omega' - \omega} d\omega'\end{aligned} \quad (3).$$

We perform the K-K transformation with assuming $\mathrm{Im}\Sigma_{ep}$ to be a constant for $|\omega|=|E_B|>100$ meV as previous practices[5,6], since the K-K transformation of a constant is zero according to Eq. (3). In this work, we assign this constant to be an averaged value of $\mathrm{Im}\Sigma_{ep}$ at E_B ~ [60, 100] meV. As shown in Figs. S1c-d, using the self-energies of the α and β bands of CsV_3Sb_5 as examples, the consistency of the extracted self-energy is confirmed by the good consistency between the experimental $\mathrm{Re}\Sigma$ and the K-K transformations of the extracted $\mathrm{Im}\Sigma_{ep}$. This good consistency validates the bare band assumption.

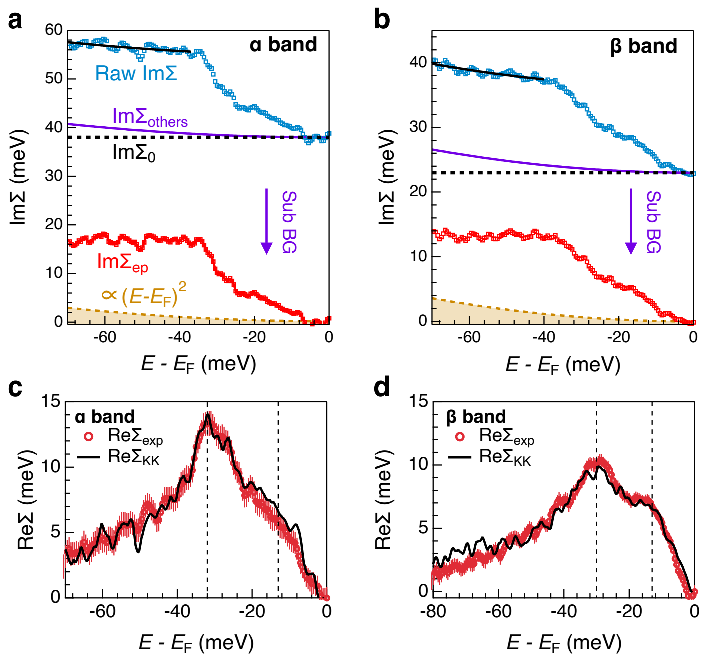

Fig. S1. Background subtraction and consistency-check of the self-energy following Kramers-Kronig relation. a, Raw imaginary-part self-energy ImΣ of the α band derived from the FWHMs by multiplying bare velocity v_0 of 3.824 eV/Å. **b**, ImΣ of the β band derived from the FWHMs by multiplying v_0 of -3.887 eV/Å. $\mathrm{Im}\Sigma_{ep}$ in **a-b** are extracted after subtracting $\mathrm{Im}\Sigma_{others}$ as described in supplementary note 2. **c-d**, Experimentally extracted real-part self-energy ReΣ (circle marks) for the α and β bands, respectively. The black lines in **c-d** are $\mathrm{Re}\Sigma_{KK}$ transformed from the $\mathrm{Im}\Sigma_{ep}$ following Kramers-Kronig relation (Eq. (3)). The α and β band here are the ones presented in Fig. 2 of the main text.

4. Comparison of the Eliashberg function extracted from the different fit procedures

To examine the validity of the extracted Eliashberg function $\alpha^2F(\omega)$, in Fig. S2 we compare the $\alpha^2F(\omega)$ from the ReΣ and ImΣ via the maximum entropy method (MEM) for the α and β bands of CsV_3Sb_5. For simplicity, we extract $\alpha^2F(\omega)$ from ImΣ' with a constant background of $\mathrm{Im}\Sigma_0$ subtracted, since $\mathrm{Im}\Sigma_{ep} \simeq \mathrm{Im}\Sigma' = \mathrm{Im}\Sigma - \mathrm{Im}\Sigma_0$ with $E_B < 60$ meV (Figs. S1a-b). As shown in Fig. S2d, the extracted $\alpha^2F(\omega)$ overlap well except for a deviation near 12 meV on the α band. This could be explained by that the strength of the 12-meV kink is much weaker compared to the 32-meV kink on the α band thus the uncertainties of the MEM fits are increased. We count this deviation into the error bars of the determined EPC strength.

As a crosscheck, we also extracted the $\alpha^2F(\omega)$ from a self-consistent procedure: (1) we assume an initial trial v_0 and calculate the ReΣ and ImΣ' from the experimental band dispersions according to Eqs. (1)-(2); (2) deduce the $\alpha^2F(\omega)$ from the ImΣ' via MEM; (3) calculate a $\mathrm{Re}\Sigma^*$ from the $\alpha^2F(\omega)$ following Eq. (1) described in methods of the main text. By changing the v_0 and

repeating steps (1)-(3), we can eventually find a proper v_0 to minimize the difference between the ReΣ and ReΣ*. We note that the above self-consistent procedure is to find out the self-consistent ReΣ and ImΣ' linked through the K-K relation by tuning v_0. We performed such fits for the α and β bands of CsV_3Sb_5 (Fig. S2c). The fitted v_0 is highly consistent with the assumed bare velocity of the linear dispersion connecting the E_B~100 meV and the E_F as shown in Table S1, and the extracted $\alpha^2F(\omega)$ is also consistent as shown in Fig. S2d.

More importantly, the EPC strength λ and Debye temperature θ_D estimated from these three fits have a good consistency, as shown in Fig. S2e. These all validate the extracted $\alpha^2F(\omega)$ and the bare band assumption. In the main text and the later estimation of MacMillan's superconducting transition temperature, we used the $\alpha^2F(\omega)$ extracted from the fits of the ReΣ based on the assumed v_0.

Table S1. Comparison of the bare velocities for the α and β band shown in Fig. 2.

band	Fitted v_0 (eV/Å)	assumed v_0 (eV/Å)	difference
α	3.950	3.824	3.2%
β	-3.929	-3.887	1.1%

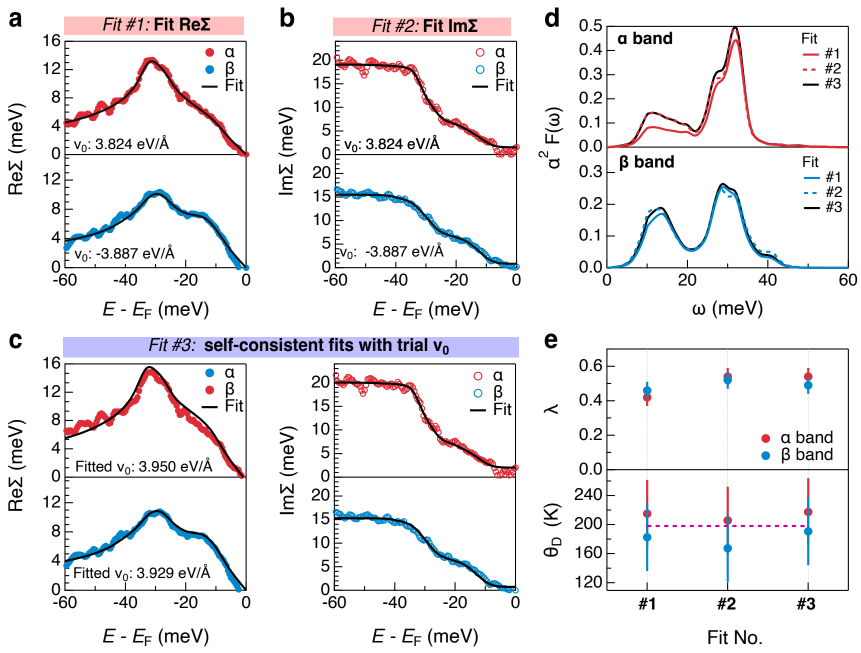

Fig. S2. Comparison of the Eliashberg function $\alpha^2F(\omega)$ from different fit procedures. a, Real parts of the self-energy ReΣ and the corresponding fits to extract the $\alpha^2F(\omega)$ (fit #1). **b,** Imaginary parts of the self-

energy $\text{Im}\Sigma' = \text{Im}\Sigma - \text{Im}\Sigma_0$ and the corresponding fits to extract the $\alpha^2F(\omega)$ (fit #2). The ReΣ and ImΣ' in **a** and **b** are extracted based on the assumed bare velocity v_0. **c,** Self-consistent fits on the experimental band dispersions with the trail bare velocities (fit #3). **d,** Comparison of the $\alpha^2F(\omega)$ extracted from the fits #1, #2, and #3. **e**, Estimated EPC strength λ and Debye temperature θ_D from the fits #1, #2, and #3. The α and β bands in this figure are the ones shown in Fig. 2 of the main text.

5. Electronic kinks over momentum space

To investigate the momentum dependence of the EPC, we took 8 cuts along the FS of the α band and 9 cuts along the FS of the β band as shown in Fig. S3c. The ARPES intensity plots of these cuts are shown in Fig. S3a and S3b. Through applying Lorentzian fits to the MDCs, the extracted band dispersions are summarized in Figs. S3d, e for the α bands, and Figs. S3h, i for the β bands. The deviations of the band dispersions at higher E_B to the linear fits at lower E_B highlight the electronic kinks. The kink energies can be determined by the peak positions of the ReΣ(ω) plots shown in Figs. S3g and S3k. Clearly, the kink energies are momentum independent. The λ_{dev} is obtained by a linear fit at an energy range smaller than E_B~12 meV. The fits to determine λ_{dev} presented in Fig. 3f of the main text are demonstrated as black lines in Figs. S3g and S3h.

It is worth mentioning the momentum- and energy-dependent EPC on the α and β bands captured by analyzing the change ratio $(v_b/v_a - 1)$ between the band velocities below (v_b) and above (v_a) the kink energy. As shown in Fig. S3f, both the 12-meV kink and 32-meV kink are nearly momentum-independent while the situations on the β bands are different. As shown in Fig. S3j, the 12-meV kink on the β-band becomes weaker from the Γ-K direction to the Γ-M direction in contrast to the 32-meV kink which shows the opposite behavior and becomes stronger approaching to the Γ-M direction. The EPC vertex generally shows energy and momentum dependence. It can be strongly affected by the symmetry of the phonon mode, the orbital character of the electronic bands, the electronic density of states, *etc*. For the *d*-electron, both the orbital character and the electronic density of states show a strong momentum dependence, and therefore momentum-dependent EPC is expected. Interestingly, the opposite evolutions of the 12-meV and 32-meV kinks on the β-band yield a nearly momentum-independent λ (Fig. 3 of the main text).

We shall note the data showing in Fig. S3 or Fig. 3 of the main text were collected from a sample (denoted as "sample 1") which has a small side-peak nearby the main peak in the MDCs

of the α band. This side-peak could be induced by the multiple "CDW domains" within our laser spot size (~50 μm) since the measurements were performed in the CDW phase. We confirm that this side peak is too weak to affect the MDCs' fittings. As shown in Figs. S4a-b, the FWHM of the fitted MDCs is the same with a "domain-free" sample (denoted as "sample 2") of which the MDCs can be fitted well by a single-peak Lorentzian. Importantly, as shown in Figs. S4c-f, the momentum distribution of λ_{dev} for independent samples 1 and 2 are highly consistent, confirming the robustness of our conclusion. The data for demonstrating the electronic kinks in Figs. 1d-e of the main text were collected from sample 2.

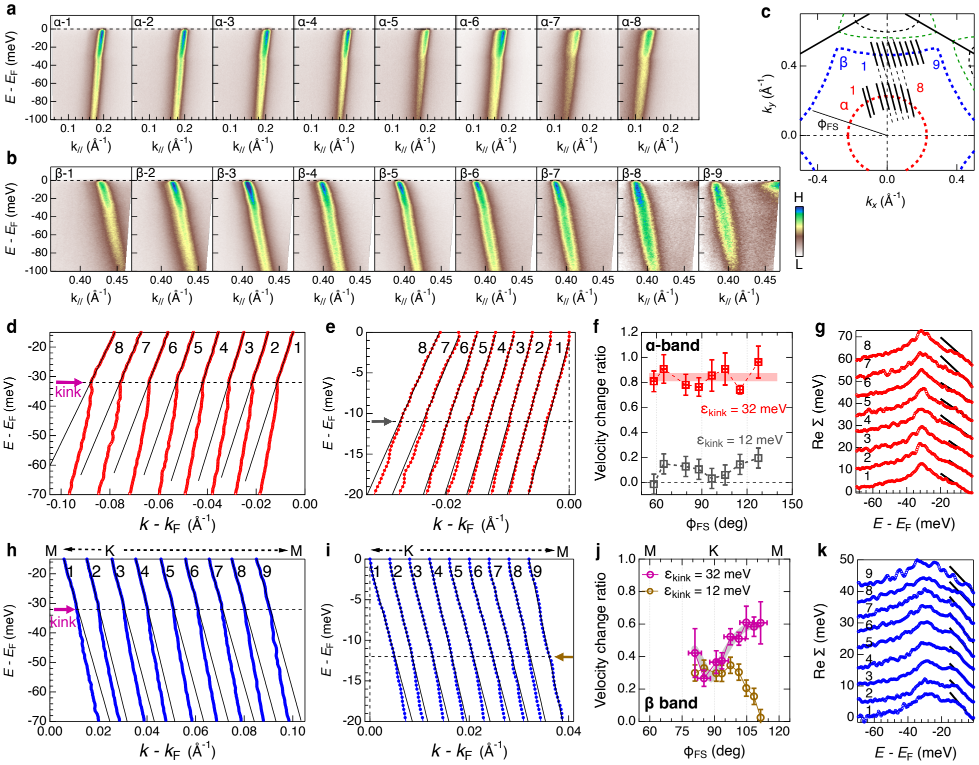

Fig. S3. The kink dispersion over momentum space of CsV_3Sb_5. a-b, ARPES intensity plots of the α and β bands taken along their FSs as shown in **c**. **d-e,** Band dispersions of the α bands shown in binding energy range [15, 70] meV and [0, 20] meV, respectively. The black lines are the linear fits to band dispersions at energy range of [-15, -30] meV in **d,h** and at energy range of [-9, 0] meV in **e, i**. **f,** Change ratio between the band velocities below and above the kinks. **g,** ReΣ(ω) plots of the α bands. The black lines are the linear fits to determine λ_{dev}. **h-k,** Same with **d-g** but for the β bands.

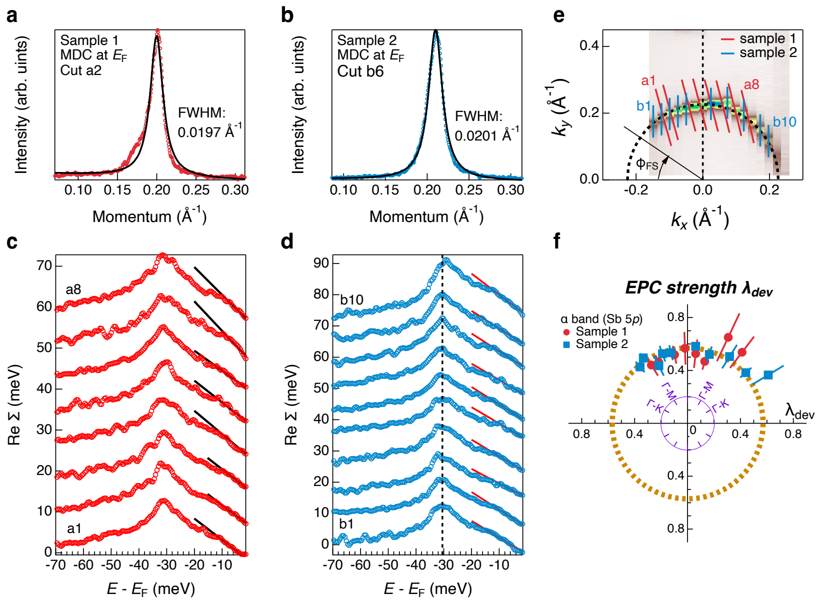

Fig. S4. Repeat measurements for the kinks on the α band. a MDCs at E_F on sample 1. **b** Same with **a** but on sample 2. The black lines are the single-peak Lorentzian fits. **c,** Real-part self-energies ReΣ on sample 1 for the cuts shown in **e**, on which the Fermi surface mapping of sample 2 is appended. **d,** Same with **c** but for sample 2. **f,** Comparison of the EPC strength λ_{dev} measured on the samples 1 and 2.

6. Estimation of McMillan's superconducting transition temperature

The superconducting transition temperature T_c is estimated based on McMillan's formula[7]:

$$T_c = \frac{\theta_D}{1.45} \exp\left[-\frac{1.04(1+\lambda)}{\lambda-\mu^*(1+0.62\lambda)}\right] \qquad (4).$$

Here θ_D is the Debye temperature, λ and μ^* are dimensionless parameters that describe the strength of effective electron-electron attractive and screened-repulsive interactions, respectively. When $\lambda > \mu^*$, the superconductivity can happen.

Estimated T_c for the pristine CsV_3Sb_5. The Debye temperature θ_D is the average frequency of the coupled phonon modes calculated from the Eliashberg function $\alpha^2F(\omega)$ according to Eqs. (2)-(3) in main text. The average frequencies for the α and β bands are ~18.5 meV and ~15.7 meV, respectively. We take the average value ~17.1 meV to calculate the T_c driven by EPC. Thus, θ_D equals ~198 K. The parameter μ^* is assigned to be 0.12, which is a theoretically suggested value[2]. Based on the θ_D = 198 K and μ^* = 0.12, the λ=0.45 - 0.6 determined from the kinks in the electronic band structure gives a T_c from 0.8 K ~ 3 K. The upper limit is close to the experimental T_c of the CsV_3Sb_5 sample (Table S2).

Estimated T_c for the $Cs(V_{0.93}Nb_{0.07})_3Sb_5$. Since the EPC on the β band is larger than that on the α band, the EPC on the β band is dominated in $Cs(V_{0.93}Nb_{0.07})_3Sb_5$. Here we use the parameters from the β band to estimate the maximum T_c driven from EPC. The average frequency of the coupled phonons is ~11.4 meV, corresponding to a Debye temperature of ~132 K. Since the Nb substitution does not change the carrier density, the μ^* still equals 0.12 Considering the $\lambda \sim 0.75 \pm 0.05$, the T_c is estimated to be from 3.2 K to 4.5 K. The upper limit is also close to the experimental T_c of $Cs(V_{0.93}Nb_{0.07})_3Sb_5$ (Table S2).

Table S2. Experimental parameters for the estimation of the Macmillan's T_c.

sample	θ_D(K)	λ	$T_c^{McMillan}$(K)	T_c^{exp}(K)
CsV_3Sb_5	198	0.45 ~ 0.6	0.8 ~ 3	2.6
$Cs(V_{0.93}Nb_{0.07})_3Sb_5$	132	0.75 ± 0.05	3.2 ~ 4.5	4.4

7. Electronic kinks and charge density wave gaps on the δ band of CsV_3Sb_5

Both β and δ bands are captured in the cuts marked in Fig. S6a. The ARPES intensity plots along cut 1 and cut 2 are shown in Figs. S6b and S6c. The V $3d_{yz}$ orbital-derived β bands are favored in intensity under *s*-polarized light while the V $3d_{x^2-y^2}$ orbital-derived δ bands are favored in intensity under *p*-polarized light. As the extracted band dispersions in Fig. S6d and the ReΣ(ω) plots in Fig. S6e, the kinks at E_B~12 meV and 32 meV are also observed on the δ band.

By symmetrizing the EDCs at k_F shown in Fig. S6f, a charge density wave (CDW) gap, which manifests as a dip at E_F, opens on the δ bands but is absent on the α and β bands (Fig. S5). The CDW gap becomes larger on the δ band when approaching the M point, which makes the band flatter at E_F, as Figs. S6b and S6c showing. This flatness hinders the precise estimation of the EPC strength.

As the phase diagram shown in Fig. 1a of the main text, the superconductivity and CDW are competing orders in CsV_3Sb_5, hence it is expected to yield an anisotropic superconducting (SC) gap symmetry, which seems to contradict the isotropic SC gap symmetry expected by EPC mechanism. Our understanding of this puzzling issue is related to the multi-orbital degrees of freedom. As shown in previous DFT calculations[2] of AV_3Sb_5, there are multiple van Hove singularities near E_F. Depending on the types and binding energies of these van Hove singularities,

the CDW gap will be very different. Actually, due to the absence of particle-hole symmetry of the CDW phase, even the center erengy of the CDW gap can be away from the E_F. Therefore, the electronic density of state at E_F is not expected to be a clean gap as confirmed by the ARPES measurements (Fig. S6f). In this case, the SC gap near the M-point can be induced by the pair-hopping term. Consequently, a nearly isotropic SC gap function can be realized.

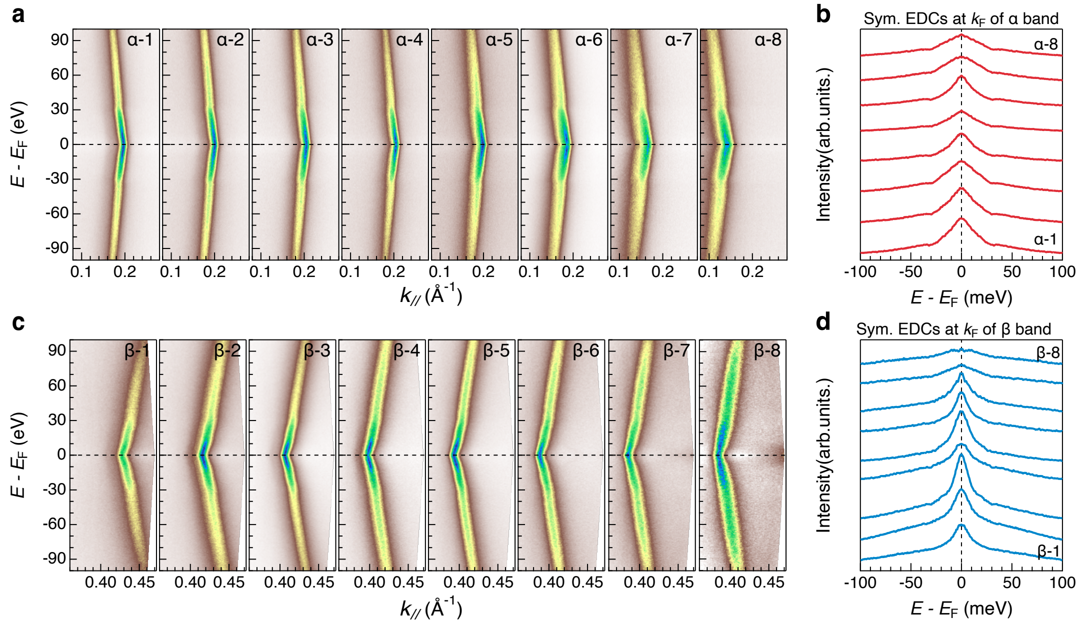

Fig. S5. Symmetrized EDCs for the α and β bands of CsV_3Sb_5. a, Symmetrized ARPES intensity plots for the α bands as the cuts shown in Fig. S3c. **b**, Symmetrized EDCs at k_F of the α bands. **c-d**, Same with **a-b** but for the β bands as the cuts shown in Fig. S3c.

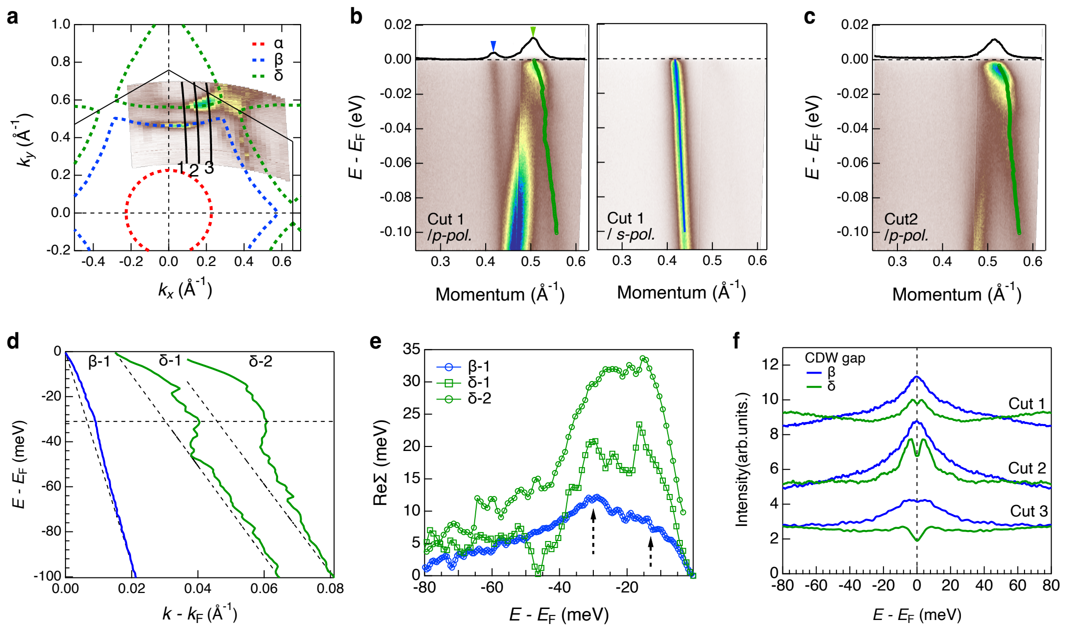

Fig. S6. The kink dispersion and CDW gap in the δ bands of CsV_3Sb_5. a, ARPES intensity integrated over E_F ± 10 meV. The dashed lines represent the Fermi surface contours. **b-c,** ARPES intensity plots of cut 1 and 2 marked in **a** measured with *s*- or *p*-polarized light. The black line in left panel of **b** is the MDCs at E_F which shows two peaks contributed from the β and δ bands. The green dotted lines are the dispersions derived from fits to the MDCs. **d,** Extracted band dispersions of the β and δ bands in cut 1 and the δ band in cut 2. Bare bands are shown as black dashed lines. **e,** ReΣ(ω) of the band dispersion presented in **d**. **f,** Symmetrized EDCs at k_F of the β and δ bands along cut 1 to cut 3 marked in **a**.

8. Persist electronic kink with temperature increasing

We claim that the observed electronic kinks origin from the EPC because that these electronic kinks are persist with temperature and even above T_{CDW} ~ 94 K, as the ARPES spectra shown in Figs. S7a-b and the ReΣ(T) plots shown in Figs. S7c-d. We shall point out that the 32-meV kink on the β band looks like less prominent compare with the one on the α band at higher temperature. This can be understood by the different coupling strengths of the 12-meV and 32-meV kinks on the α and β bands as shown in Fig. 2 of the main text. On the β band these two kinks have the comparable strengths at the low temperature, with the temperature increasing, the dip in the ReΣ(T) plots between two peaks contributed from the 32-meV kink and the 12-meV kink is filled due to the broadness (Fig. S7d), which makes the 32-meV kink look like less prominent. Whereas, on the α band the 12-meV kink is much weaker than the 32-meV kink at the low temperature, thus the 32-meV kink is still prominent at higher temperature despite of the broadness.

To examine the temperature-dependent EPC, we show the extracted $\lambda_{dev}(T)$ in Fig. S7e. Due to the temperature-dependent electron-phonon scatterings, $\lambda_{dev}(T)$ decreases as temperature increases. Interestingly, an inflection point is observed at T_{CDW}, uncovering an intimate correlation between the CDW and EPC in CsV_3Sb_5. We also shall note, however, $\lambda_{dev}(T)$ is a qualitative estimation of the temperature-dependent EPC strength since $\mathrm{Re}\Sigma(T)$ is acquired here by assuming a temperature-independent linear bare band (dashed lines in Fig. S7b). Moreover, as discussed in the main text, $\lambda_{dev} \simeq \lambda$ is valid only when $T << \theta_D$.

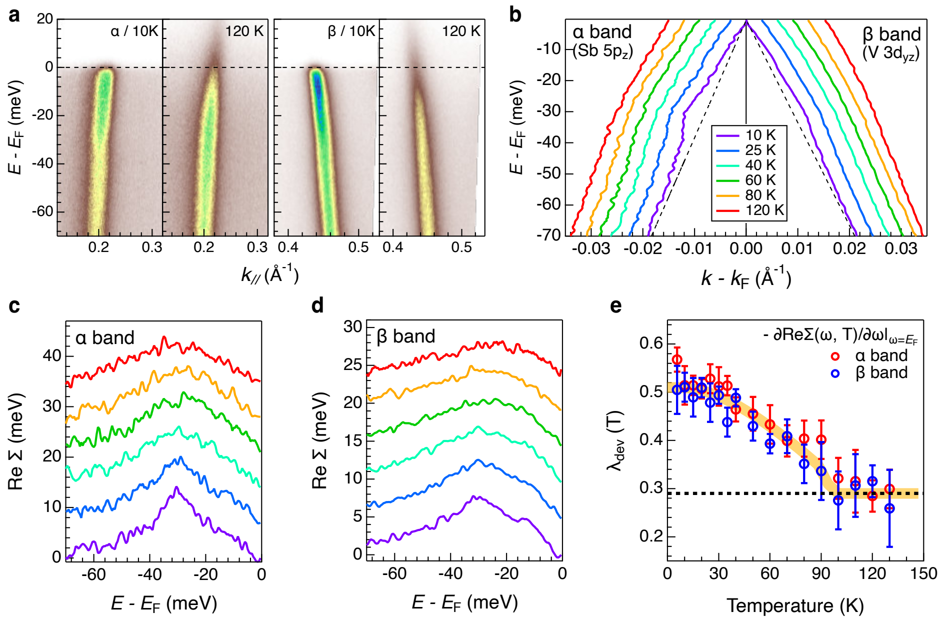

Fig. S7. Persistent kinks above CDW transition temperature at 94 K in CsV_3Sb_5. a, ARPES intensity plots of the α and β bands along Γ-K direction of CsV_3Sb_5 below and above T_{CDW} ~ 94 K. **b,** Extracted band dispersions at the representative temperatures. **c-d,** Temperature-dependent real part of the self-energy ReΣ for the α and β bands, respectively. **e,** Temperature evolution of the first derivative of ReΣ at E_F for the α and β bands.

Supplementary References

1 Sobota, J. A., He, Y. & Shen, Z.-X. Angle-resolved photoemission studies of quantum materials. *Reviews of Modern Physics* **93**, 025006 (2021).

2 Tan, H., Liu, Y., Wang, Z. & Yan, B. Charge Density Waves and Electronic Properties of Superconducting Kagome Metals. *Physical Review Letters* **127**, 046401 (2021).

3 Li, C. *et al.* Coexistence of two intertwined charge density waves in a kagome system. *Physical Review Research* **4**, 033072 (2022).

4 Valla, T., Fedorov, A. V., Johnson, P. D. & Hulbert, S. L. Many-Body Effects in Angle-Resolved Photoemission: Quasiparticle Energy and Lifetime of a Mo(110) Surface State. *Physical Review Letters* **83**, 2085-2088 (1999).

5 Yu, T. *et al.* Strong band renormalization and emergent ferromagnetism induced by electron-antiferromagnetic-magnon coupling. *Nature communications* **13**, 6560 (2022).

6 Kordyuk, A. A. *et al.* Bare electron dispersion from experiment: Self-consistent self-energy analysis of photoemission data. *Physical Review B* **71**, 214513 (2005).

7 McMillan, W. Transition temperature of strong-coupled superconductors. *Physical Review* **167**, 331 (1968).
